# Continuous Feeding Insulin Injection (CFII): A New Simple Method to Stabilize Severe Glucose Variability and Nutrition Delivery in Critically Ill Patients

**DOI:** 10.7759/cureus.78758

**Published:** 2025-02-09

**Authors:** Yuzuru Ohshiro

**Affiliations:** 1 Department of Internal Medicine, Omoromachi Medical Center, Naha, JPN

**Keywords:** continuous glucose monitoring systems, continuous subcutaneous insulin infusion therapy, critically ill patients, diabetes mellitis, early nutrition, efficacy of intravenous insulin infusion, enteral and parenteral nutrition, hypoglycemia, medical intensive care unit (micu), s: sepsis

## Abstract

This report describes a new, simple, and systematic approach, Continuous Feeding Insulin Injection (CFII), designed to stabilize severe glucose variability and optimize nutritional delivery in critically ill patients when intensive insulin therapy (IIT) alone proves insufficient. Septic patients frequently develop inflammation-induced insulin resistance, resulting in recurrent episodes of hyperglycemia and hypoglycemia. Fever, systemic inflammation, and appetite loss further disrupt glucose homeostasis, complicating nutritional management. CFII integrates two key components: continuous enteral nutrition (CEN) delivered at a fixed rate over 24 hours, based on the patient’s metabolic needs, and continuous insulin infusion dynamically adjusted according to frequent or continuous blood glucose monitoring. This approach stabilizes both blood glucose levels and nutritional intake.

We present a case of a 65-year-old woman with type 2 diabetes (BMI 21.2 kg/m²) who developed sepsis secondary to pyelonephritis. Despite receiving intensive insulin therapy (IIT), she experienced severe glycemic fluctuations (38-361 mg/dL; mean±SD: 218.6±110.0 mg/dL) and recurrent hypoglycemia, rendering oral intake nearly impossible. CFII was initiated with enteral feeding starting at 1000 kcal/day and gradually increased to 1400 kcal/day, while insulin infusion was dynamically adjusted every three hours. This strategy successfully stabilized severe glucose variability (164.5±35.9 mg/dL), eliminated hypoglycemic episodes, and achieved controlled nutrition delivery.

Clinically, similar approaches are presumed to have been used in patients receiving CEN; however, this is the first report to the best of our knowledge to systematically describe CFII as a structured method for glucose and nutritional management in critically ill patients, to propose the term "CFII" and to demonstrate its effectiveness in a patient for whom IIT alone was insufficient. CFII enables nutritional delivery to be tailored to the course of treatment while maintaining stable glycemic control. Its simplicity, practicality, and compatibility with existing hospital systems make CFII an accessible method for broader clinical application. CFII has the potential to improve metabolic outcomes and enhance survival rates in this vulnerable patient population. Further research, including systematic evaluations and randomized controlled trials, is necessary to confirm its efficacy, safety, and applicability across diverse clinical settings.

## Introduction

Hyperglycemia and glucose variability are significant challenges in critically ill patients, particularly those with sepsis or septic shock. The latest Society of Critical Care Medicine (SCCM) and 2021 Surviving Sepsis Campaign (SSC) guidelines emphasize the importance of glycemic control, recommending insulin therapy initiation when blood glucose levels exceed 180 mg/dL, with a target range of 144-180 mg/dL to balance optimal glucose management while minimizing hypoglycemia risk. Despite these recommendations, achieving stable glycemic control remains difficult due to the dynamic metabolic changes inherent in critical illness [1].

Intensive insulin therapy (IIT) has traditionally been the cornerstone of glycemic management in critically ill patients, demonstrating benefits in reducing infection rates and mortality in some studies [2]. However, the application of IIT remains controversial due to the increased risk of hypoglycemia and significant glucose variability, both of which are associated with worsened patient outcomes [3-5]. Large-scale randomized trials have shown that while IIT can achieve lower mean glucose levels, it does not necessarily translate into improved survival, particularly when hypoglycemic events occur [6]. Recent evidence suggests that continuous enteral nutrition (CEN) may be a crucial factor in achieving glycemic stability. Enteral nutrition is preferred over parenteral nutrition in critically ill patients as it preserves gut integrity, reduces infection risk, and enhances patient recovery [7-12]. However, conventional glycemic management strategies often fail to integrate insulin delivery with enteral feeding in a way that accommodates the metabolic fluctuations of critically ill patients.

Furthermore, in parenteral nutrition, both continuous subcutaneous insulin infusion and continuous intravenous insulin infusion are standard methods for glycemic control [13-16]. The potential of continuous intravenous insulin infusion to improve glycemic control in patients receiving enteral nutrition has also been suggested, highlighting the need for further research [17]. Based on this, the combination of CEN and continuous insulin infusion is also presumed to be practiced clinically. However, a comprehensive search using PubMed, Google Scholar, and Scopus did not identify any systematic reports describing the structured application of CEN combined with continuous insulin infusion for the management of nutrition and glycemic control in critically ill patients as an adaptation for critically ill patients.

To address these challenges, this report proposes Continuous Feeding Insulin Injection (CFII) as a novel and straightforward method to stabilize glucose variability while optimizing nutritional support in critically ill patients who can tolerate enteral nutrition but cannot achieve adequate glycemic control with IIT alone. CFII integrates insulin administration with continuous enteral nutrition, allowing real-time adjustments to insulin dosage based on the patient’s dynamic metabolic state. This approach not only reduces glucose variability but also ensures consistent and adequate nutrition delivery, mitigating the risks of malnutrition and metabolic imbalance. By stabilizing blood glucose levels, CFII facilitates the maintenance of nutritional support that is critical for providing the energy and substrates necessary for recovery in septic patients. CFII aligns with the principles of both SCCM and SSC by enhancing precision in glycemic control and supporting early and appropriate nutritional management [1,18]. By addressing the dual challenges of glucose variability and nutritional deficits, CFII has the potential to improve metabolic outcomes, reduce inflammation, and enhance survival rates in this vulnerable patient population.

## Case presentation

A 65-year-old woman with a 20-year history of type 2 diabetes treated with insulin was admitted to our hospital. Her regimen included twelve units of insulin glargine in the morning and four units of insulin lispro before each meal. She presented with a one-day history of fever. She was 155 cm tall, weighed 51 kg, and had a BMI of 21.2 kg/m². On admission, she was diagnosed with pyelonephritis and subsequently sepsis. Her HbA1c was 8.2%, reflecting poor glycemic control, which was thought to have contributed to the development of her condition.

Upon examination, the patient was alert (GCS 15), with a blood pressure of 98/50 mmHg, heart rate of 110/min, respiratory rate of 23/min, oxygen saturation (SpO₂) of 93%, and body temperature of 39.0°C. Laboratory tests revealed a white blood cell count of 12,000/mm³, elevated C-reactive protein (14 mg/dL), aspartate aminotransaminase (70 U/L), alanine aminitransaminase (60 U/L), and total bilirubin (1.4 mg/dL). The platelet count was reduced to 120,000/mm³. Kidney function had deteriorated compared to one month earlier (blood urea nitrogen 19.4 → 28.4 mg/dL, creatinine 1.0 → 1.6 mg/dL). Urinalysis showed pyuria and bacteriuria, with *Escherichia coli* detected. Blood cultures, obtained twice on admission, also yielded *Escherichia coli*, confirming bacteremia. Based on positive blood cultures, fever, hypotension, and organ dysfunction (worsened renal function), the patient met the Sepsis-3 criteria and was diagnosed with sepsis [1].

The patient had no history of gastrointestinal disease, and a physical examination revealed no signs of gastrointestinal dysfunction. Abdominal auscultation confirmed normal bowel sounds, and the patient reported no abdominal pain or discomfort. Abdominal ultrasound findings revealed no abnormalities in the liver or intestines, supporting the decision to initiate enteral nutrition.

Treatment included cefmetazole (1.0 g drip infusion twice daily for 10 days) and fluid replacement therapy (1 L/day). This resulted in significant clinical improvement, with blood pressure stabilizing at 110/80 mmHg and a heart rate of 90/min on the following day and 130/80 mmHg with a heart rate of 70/min the day after. Respiratory rate normalized to 16/min, SpO₂ improved to 98%, and body temperature returned to normal.

CFII method

The patient’s oral intake was estimated to be 850 kcal/day, which was considered nutritionally deficient. As the patient became unable to consume any meals, continuous enteral nutrition was initiated via a nasogastric tube using RACOL-NF liquid (Otsuka Pharmaceutical Co.). This formula, with an osmolarity of approximately 300 mOsm/L, provides 100 kcal per 100 mL, including 4.0 g protein, 3.3 g fat, and 13.8 g carbohydrates, along with essential vitamins, minerals, and trace elements. It was administered at a constant rate over 24 hours to ensure adequate nutritional support.

On the third day of hospitalization (the first day of CFII), enteral nutrition was initiated at 1000 kcal/day to avoid metabolic overload. To progressively meet the patient’s metabolic and nutritional demands, the enteral nutrition was increased to 1200 kcal/day on the fourth day of hospitalization (second day of CFII) and to 1400 kcal/day on the fifth day of hospitalization (third day of CFII). The initiation rate and adjustment method of insulin infusion are described below. In addition, the insulin infusion rate was preemptively increased by 15% on both the second and third days of CFII to align with the increased glucose load from the higher nutritional intake. Blood glucose levels were monitored every three hours using a bedside glucometer, and the insulin infusion rate was dynamically adjusted according to the following protocols:

CFII Day 1 (Hospitalization Day 3) Insulin Adjustment Protocol

The initial insulin infusion rate of 0.5 units per hour (equivalent to 12 units per day for an enteral nutrition intake of 1000 kcal per day) was determined based on the patient’s pre-sepsis oral intake of 1400 kcal per day and their insulin requirement of 18 units per day before the onset of sepsis, prior to CFII initiation. When blood glucose levels dropped below 100 mg/dL, the insulin infusion was temporarily stopped to prevent hypoglycemia. A glucose supplement of 10 g was administered intravenously, and blood glucose levels were closely monitored. When blood glucose subsequently exceeded 200 mg/dL, the insulin infusion was restarted at a rate of 0.5 units per hour. For blood glucose levels between 100 and 140 mg/dL, the infusion rate was reduced by 0.5 units per hour to avoid excessive insulin administration. When blood glucose was maintained within the range of 141 to 200 mg/dL, the current infusion rate was continued without modification. However, when blood glucose exceeded 200 mg/dL, the insulin infusion rate was increased by 0.5 units per hour to correct hyperglycemia.

CFII Day 2-5 (Hospitalization Day 4-7) Insulin Adjustment Protocol

As blood glucose levels began to stabilize and to accommodate the increased enteral nutrition, the insulin infusion protocol was adjusted dynamically. When blood glucose levels dropped below 100 mg/dL, the insulin infusion was temporarily discontinued, and a glucose supplement of 10 g was administered intravenously. The patient was closely monitored, and insulin infusion was restarted at 0.5 units per hour only if blood glucose levels later exceeded 180 mg/dL. For blood glucose levels below 140 mg/dL, the insulin infusion rate was reduced by 0.3 units per hour to minimize the risk of hypoglycemia. When blood glucose was maintained within the range of 141 to 180 mg/dL, the insulin infusion rate remained unchanged. However, if blood glucose exceeded 180 mg/dL, the infusion rate was increased by 0.3 units per hour to enhance glycemic control. On the second day, when the enteral nutrition was increased to 1200 kcal/day, the insulin infusion rate was preemptively increased by 15% to align with the increased glucose load. Similarly, on the third day, with a further increase to 1400 kcal/day, another 15% increase in the insulin infusion rate was implemented. These preemptive adjustments ensured that blood glucose levels remained stable despite the increased nutritional intake.

Rationale for Adjustments

The initial insulin infusion rate of 0.5 units/hour was calculated based on the patient’s prescribed dietary intake and prior insulin usage. As the nutritional intake was gradually increased, corresponding preemptive adjustments to the insulin infusion rate (15% increases on the second and third days) were made to match the increased glucose load and prevent hyperglycemia. Additionally, the reduction in insulin adjustment increments (from ±0.5 units/hour on the first day to ±0.3 units/hour on subsequent days) aimed to maintain fine control as blood glucose levels stabilized. This dynamic approach allowed for individualized management, minimizing the risks of both hypoglycemia and hyperglycemia.

Results

Pre-CFII (Intensive Insulin Therapy, Day 2-3)

**Figure 1:**
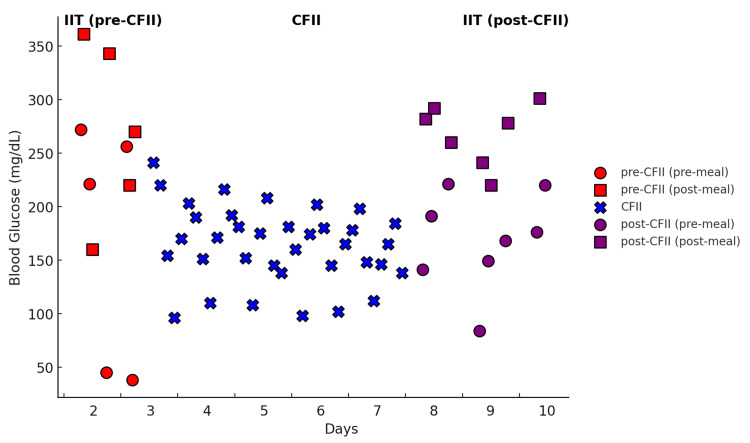
Blood glucose variability before, during, and after CFII implementation. Blood glucose levels fluctuated significantly during the pre-CFII phase (intensive insulin therapy, Day 2-3), ranging from 38 to 361 mg/dL. Following the initiation of Continuous Feeding Insulin Injection (CFII) on Day 3, blood glucose variability progressively decreased, with further stabilization from Day 4 onwards. During the post-CFII phase (Day 8-10), blood glucose fluctuations increased again, demonstrating a return to the pre-CFII instability pattern. CFII was effective in reducing glucose variability and preventing hypoglycemia.

During the initial intensive insulin therapy, the patient’s blood glucose (BG) levels were highly variable, with extreme fluctuations ranging from severe hyperglycemia (361 mg/dL post-meal) to hypoglycemia (38 mg/dL pre-meal) (Figure 1).

**Figure 2:**
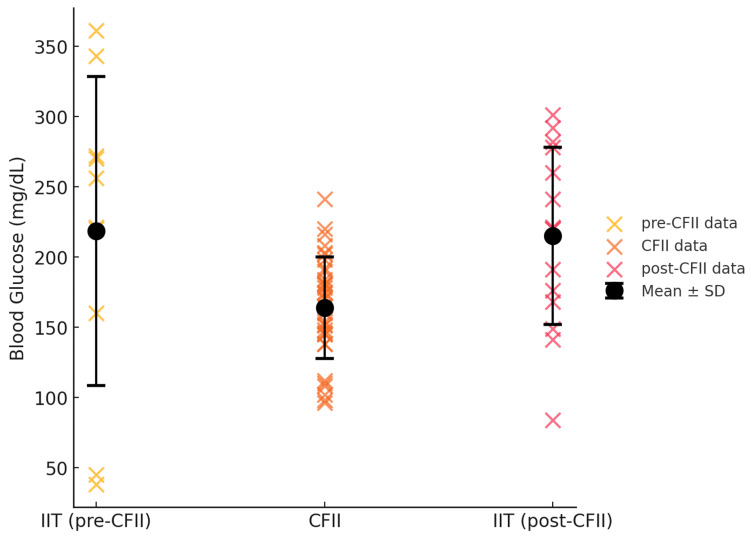
Mean blood glucose levels and standard deviation across treatment phases. The mean±SD blood glucose levels during the pre-CFII phase (intensive insulin therapy, Day 2-3) were 218.6±110 mg/dL, indicating poor glycemic control. With the implementation of CFII (Day 3-7), mean blood glucose levels improved to 164.5±35.9 mg/dL, with further stabilization from Day 4 onwards (162.8±31.7 mg/dL). In the post-CFII phase (Day 8-10), mean blood glucose levels increased to 214.5±60.7 mg/dL, demonstrating greater variability and reinforcing the efficacy of CFII in stabilizing glucose levels compared to conventional insulin therapy.

The mean±SD of blood glucose during this period was 218.6±110 mg/dL, demonstrating poor glycemic control (Figure 2). This variability posed a significant challenge to maintaining stable blood glucose levels and adequate nutritional intake. 

CFII (Day 3-7)

Following the initiation of CFII on Day 3, blood glucose variability progressively decreased (Figure 1). Across the entire CFII period (Day 3-7), the mean±SD blood glucose level was 164.5±35.9 mg/dL, demonstrating improved glycemic control compared to the pre-CFII phase. From Day 4 onwards (Day 4-7), blood glucose variability showed a further tendency to stabilize, with a mean±SD of 162.8±31.7 mg/dL (Figure 2). This indicates that glycemic control became progressively more stable as the CFII protocol continued. The insulin infusion rate during CFII was dynamically adjusted, ranging from 0 to 2 units/hour, based on frequent blood glucose monitoring and the patient’s nutritional intake. Dynamic insulin adjustments and preemptive increases in the infusion rate aligned with incremental increases in nutritional intake (from 1000 kcal/day on Day 1 to 1400 kcal/day on Day 3). Importantly, no episodes of hypoglycemia were observed during CFII, demonstrating its safety and effectiveness.

Post-CFII (Intensive Insulin Therapy, Day 8-10)

At the beginning of the post-CFII phase, the patient’s appetite had recovered, allowing for a return to a regular oral diet of 1400 kcal/day. The patient’s original insulin regimen (six units of insulin glargine before breakfast and four units of insulin lispro before each meal) was reinstated. The patient adhered well to the prescribed diet. However, during this phase, blood glucose variability increased again, with pre-meal and post-meal BG values fluctuating between 84-301 mg/dL (Figure 1). The mean±SD blood glucose level during this period was 214.5±60.7 mg/dL (Figure 2), indicating a significant increase in variability compared to the CFII phase. This phase demonstrated a return to the pre-CFII pattern of instability, reinforcing the efficacy of CFII in maintaining stable blood glucose levels compared to conventional insulin therapy.

Comparison of CFII (After Nutritional Increase to 1400 kcal/day) and Post-CFII

During CFII, after the nutritional intake was increased to 1400 kcal/day, the required insulin dose was 20.1 units/day, dynamically adjusted based on blood glucose levels. In contrast, during the post-CFII phase, when the patient resumed a regular oral diet of 1400 kcal/day, the total insulin requirement increased to 18 units/day. For the same caloric intake, insulin requirements were lower during CFII, and blood glucose fluctuations were smaller and more stable compared to the post-CFII phase. These findings indicate that CFII provides better glycemic control with less insulin requirement, even with the same caloric intake.

Nutritional Intake and Metabolic Response

Enteral nutrition was initiated at 1000 kcal/day on Day 1 and progressively increased to 1400 kcal/day by Day 3. This adjustment was well-tolerated, with no evidence of metabolic overload or gastrointestinal dysfunction. The preemptive 15% increases in the insulin infusion rate on Days 2 and 3 effectively matched the increased glucose load, ensuring metabolic balance and maintaining stable glycemic control throughout the CFII period.

Outcomes

Her renal function recovered to baseline levels, and liver enzyme levels normalized. The patient was provided with 1,400 kcal/day, consistent with her outpatient nutritional prescription. Blood glucose levels were measured before each meal and two hours after eating.

## Discussion

This is the first report, to the best of our knowledge, demonstrating that CFII effectively stabilizes both nutritional delivery and blood glucose levels in a critically ill patient for whom IIT was inadequate. CFII also enables nutritional delivery to be tailored to the course of treatment. Its simplicity, practicality, and compatibility with existing hospital systems make CFII an accessible method for broader clinical application. Further research is warranted to confirm its effectiveness in diverse clinical settings.

This study highlights the potential of CFII as a simple approach for managing severe glucose variability while ensuring adequate nutritional delivery in critically ill patients. Unlike conventional IIT, which often fails to address the dynamic metabolic needs of septic patients [3-5], CFII provides an integrated solution by combining continuous enteral nutrition with frequent, dynamic insulin adjustments. This dual approach not only stabilizes blood glucose levels but also ensures consistent caloric intake, which is critical for recovery in critically ill patients. The findings align with recent evidence supporting early enteral nutrition (EEN) as an essential component of sepsis management, as described in a recent systematic review [19].

The results from this case demonstrate that CFII significantly reduced glucose variability and eliminated episodes of hypoglycemia, even as nutritional intake was gradually increased. The ability to maintain stable blood glucose levels with a comparable insulin dose to conventional therapy highlights the efficiency of CFII. These findings are consistent with the principles of the 2021 SSC guidelines [1], emphasizing the importance of personalized glycemic control and early enteral nutrition in septic patients. Recent studies have suggested that EEN reduces days of mechanical ventilation and improves Sequential Organ Failure Assessment (SOFA) scores, further supporting the need for structured nutritional interventions in sepsis [7-11].

CFII’s practicality lies in its simplicity as it uses readily available hospital equipment and protocols, making it accessible for widespread clinical application. Additionally, its compatibility with existing systems means it can be implemented with minimal changes to routine hospital workflows. This ease of implementation is critical given the variability in ICU protocols across different institutions. Furthermore, studies have demonstrated that structured nutritional strategies reduce the risk of hypoglycemia and other metabolic complications associated with conventional IIT [5,6,9,13-16,20].

However, frequent blood glucose monitoring and insulin adjustments in CFII lead to an increased demand for nursing care and higher healthcare costs. These challenges highlight the negative aspects of CFII. If continuous glucose monitoring (CGM) is available, its partial implementation could reduce the burden on nursing staff by decreasing the frequency of manual blood glucose measurements. Additionally, it could contribute to the efficiency of insulin adjustments, potentially improving feasibility in clinical practice.

This report is limited by its single-patient focus, necessitating larger studies to validate CFII’s effectiveness across diverse patient populations and settings. Future research should include randomized controlled trials (RCTs) comparing CFII with standard IIT to evaluate long-term outcomes, safety, and scalability. Additionally, investigations into the impact of CFII on inflammatory markers, metabolic flexibility, and patient recovery rates would be beneficial [7,8,10].

## Conclusions

CFII could represent a simple and effective approach to stabilize glucose variability and optimize nutritional delivery in critically ill patients. This method addresses the limitations of conventional IIT by integrating continuous enteral nutrition and dynamic insulin adjustment. In this case, CFII demonstrated significant improvements in glycemic control and nutritional management, with no episodes of hypoglycemia and reduced glucose variability. Its accessibility, practicality, and alignment with existing guidelines suggest that CFII has broad potential for clinical application. Further studies are required to confirm its efficacy and safety in a wider range of clinical scenarios, but CFII offers a promising tool for managing complex metabolic challenges in critically ill patients.
